# Sonographically Detectable Idiopathic Axillary Web Syndrome With Concurrent Thrombophlebitis: A Case Report

**DOI:** 10.7759/cureus.77024

**Published:** 2025-01-06

**Authors:** Ashley Cardona, Ashley M Fernandini-Soto, Noel A Dastas-Mendez, Vincent F Carfagno, Imtiaz Ahmed

**Affiliations:** 1 Radiology, Universidad Autónoma de Guadalajara School of Medicine, Guadalajara, MEX; 2 Radiology, Universidad Autónoma de Guadalajara School of Medicine, Guadalajara, USA; 3 Radiology, Arnot Health, Elmira, USA; 4 Radiology, Tempe St. Luke's Hospital, Tempe, USA

**Keywords:** alnd: - axillary lymph node dissection, axillary web syndrome, colored flow doppler ultrasound, msk radiology, superficial thrombophlebitis

## Abstract

Axillary web syndrome (AWS), also known as Mondor Disease, is a self-limiting condition characterized by axillary pain radiating down the anteromedial upper extremity with an associated decrease in shoulder abduction and overlying palpable cords. AWS is commonly seen in female patients with a history of axillary clearance (AC), though it is also associated with patients who have undergone sentinel lymph node biopsy (SLNB), mastectomy, or radiation therapy to the axilla or breast. AWS is a significant cause of morbidity in such patients. While the exact pathophysiology is not entirely understood, AWS is thought to arise from a complex interplay of disruptions in lymphatic flow and connective tissue fibrosis following the interventions described. We present a unique case of AWS with concurrent thrombophlebitis in a 25-year-old male who presented to the ED with a two-day history of right axillary pain and no past medical or surgical history. On physical examination, the patient showed decreased range of motion (ROM) in abduction of the right upper extremity (RUE) with an overlying palpable cord-like structure at the proximal medial RUE. Following a Doppler ultrasound (US), a diagnosis of AWS with concurrent superficial thrombophlebitis was made. This case highlights the importance of considering AWS in patients without a suggestive medical history, along with the potential utility of US in approaching a diagnosis.

## Introduction

Axillary web syndrome (AWS) is a common complication following axillary lymph node dissection (ALND), also known as axillary clearance (AC), a procedure commonly utilized in the management of breast cancer, with literature reporting an incidence rate of 48.3-72% in patients with a history of AC [1, 2]. AWS is also seen in patients who have had a sentinel lymph node biopsy (SLNB), a procedure used for breast cancer staging, as well as in those who have undergone mastectomy and radiation therapy to the axilla and breast [3, 4]. The incidence of AWS has also been shown to be higher in females, patients with a history of hypertension and diabetes mellitus, those with a BMI less than 25 kg/m^2^, and those younger than 60 years [2].

This report describes an acute presentation of AWS with concurrent thrombophlebitis in a 25-year-old male patient with a BMI less than 25 kg/m^2^ and no past medical or surgical history. The patient presented to the ED with proximal anteromedial right upper extremity (RUE) pain and axillary discomfort. The physical examination revealed decreased range of motion (ROM) in abduction and the presence of a palpable cord-like structure at the proximal medial RUE. A Doppler ultrasound (US) was performed, revealing a cord-like fascial structure. Multiple thrombosed veins in the anteromedial brachial region, with an absence of visible Doppler flow, were also visualized, consistent with a diagnosis of superficial thrombophlebitis. The patient was treated with conservative measures including non-steroidal anti-inflammatory drugs (NSAIDs) and physical therapy.

## Case presentation

We present a 25-year-old male patient who presented to the ED with a 2-day history of proximal RUE and axillary pain, accompanied by decreased ROM. The patient first noticed his symptoms while attempting to raise his RUE at home and stated he was suddenly unable to raise his arm over his head. He also noticed the presence of a palpable, cord-like structure at the right axillary region at that time, which was not visible upon inspection. The patient reported no past medical or surgical history, medication or supplement use, or history of illicit substance or tobacco use. He did note a history of social alcohol consumption.

**Figure 1:**
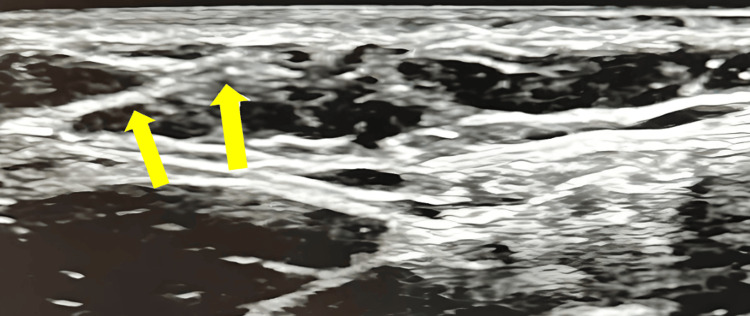
Ultrasound of the right brachial region, medial proximal aspect, demonstrating the presence of an echogenic cord-like fascial structure.

**Figure 2:**
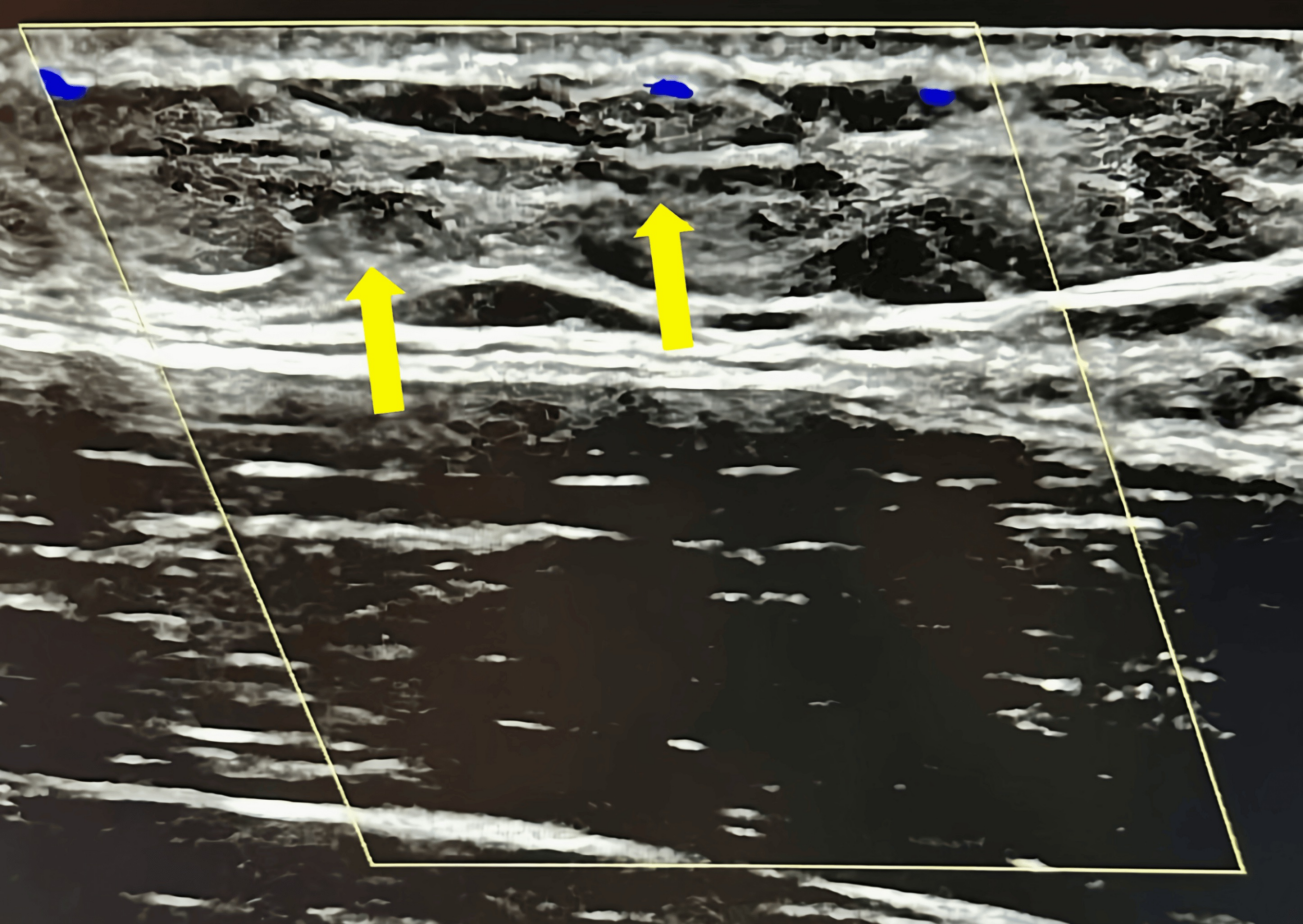
Doppler ultrasound of the right brachial region, medial proximal aspect, demonstrating an echogenic cord-like superficial thrombosed vein with an absence of visible Doppler flow.

Upon initial evaluation, the patient’s vital signs were within normal limits. The physical examination revealed decreased ROM in abduction of the RUE and the presence of a palpable cord-like structure extending from the right axilla to the right antecubital fossa. The rest of the patient’s physical exam was otherwise unremarkable. Given these findings, suspicion for AWS and superficial thrombophlebitis was raised, prompting further evaluation with Doppler US imaging of the RUE. The imaging revealed a cord-like fascial structure at the medial proximal aspect of the right brachial region (arrows, Figure 1). Multiple echogenic cord-like superficial thrombosed veins in the medial right brachial region, with an absence of visible Doppler flow, were also visualized (arrows, Figure 2).

Deep venous structures showed no signs of acute pathology. The patient was diagnosed with AWS and superficial thrombophlebitis. Treatment was approached with conservative measures, including NSAIDs and physical therapy.

## Discussion

AWS, first described in 2001, is a relatively common complication among patients treated with AC, SLNB, mastectomy, and localized radiation therapy to the axilla and breast, treatment methods collectively utilized in the treatment of breast and lymphatic malignancies [1-4]. AWS has most commonly been associated with the early post-operative period following AC lymph node surgery, with previous studies reporting an incidence rate of 48.3% in such patients, though more recent incident rates have been estimated to be as high as 72% [1, 2, 5]. AWS has also been shown to display higher incidence rates in females, patients with a history of hypertension and diabetes mellitus, those with a BMI less than 25 kg/m^2^, and those younger than 60 years [2].

The pathophysiology of AWS is not entirely understood, but it is thought to arise from disruptions in lymphatic flow and connective tissue fibrosis following the interventions described [6]. AWS has been shown, under systematic review, to most commonly present within eight weeks post-operatively, though occurrences after this time have been characterized [3]. Symptoms include the presence of palpable and tender fibrotic bands in the axillary region, which may or may not be visible on inspection, that extend down the anteromedial aspect of the arm across the antecubital fossa [7]. In severe cases, these bands may extend distally as far as the ipsilateral thenar eminence [7]. Patients typically present with decreased ROM in abduction of the RUE, and the disease is self-limiting, usually resolving within three months without treatment [6].

The diagnosis of AWS is clinical, and suspicion should be raised in the presence of characteristic fibrotic bands along with a history of AC or axillary surgery [6]. Such history is pertinent, as disruptions in lymphatic flow and inflammatory processes following procedures such as AC and SLNB are speculated to result in the fibrotic changes characterized in AWS. The utility of US in diagnosing AWS is poorly understood. Prospective studies evaluating AWS patients with US have found a lack of interpretable sonographic findings, whereas other studies analyzing sonographically diagnosed AWS patients have visualized these subcutaneous cords to be of a vascular nature [8-9]. The unclear efficacy of US in diagnosing AWS is likely due to the poorly understood pathogenesis of AWS [9]. Further studies should look to better characterize the palpable cords of AWS sonographically.

AWS may be treated with NSAIDs, though such treatment has been reported to have no effect on achieving resolution of AWS, with their utility primarily in treating the inflammatory and pain response associated with the disease [7]. Previous studies have indicated that physical therapy is an effective measure for shortening the natural course of the disease [9].

Currently, data regarding the incidence of AWS in male patients is limited. Among those reported, patients presented with a history of strenuous physical activity or trauma [6]. In the case described, no such history was noted, suggesting an idiopathic cause. Few other cases of idiopathic AWS have been discussed in the literature [10-12]. This highlights the importance of keeping AWS in the differential diagnosis for patients presenting with symptoms of axillary pain, palpable cords, and restricted ROM in the upper extremity.

## Conclusions

The case described accentuates the importance of considering AWS in patients presenting with shoulder and axillary pain, regardless of gender, absence of surgical history, or lack of visible cord formation upon inspection. Although AWS is primarily diagnosed clinically, a better understanding of its pathophysiology may help determine the utility of US in approaching or confirming a diagnosis.
